# P-988. Evaluation of Intramuscular Penicillin Use and Treatment Outcomes in Neonatal Patients

**DOI:** 10.1093/ofid/ofae631.1178

**Published:** 2025-01-29

**Authors:** Ryan H Rochat, Ali L Goldensoph, Grant T Stimes

**Affiliations:** Baylor College of Medicine, Houston, Texas; Children's Mercy Kansas City, Chariton, Iowa; Texas Children's Hospital, Houston, Texas

## Abstract

**Background:**

The prevalence of syphilis in the United States has been increasing despite available treatment options. Penicillin (PCN) is the cornerstone of syphilis treatment, and patients who do not have evidence of central nervous system involvement are often recommended a single dose of intramuscular (IM) PCN as treatment. At this time, IM PCN is on shortage nationwide, and is estimated to continue throughout 2024. The purpose of this study was to identify current length of stay trends for neonatal patients who are treated with a single dose of IM PCN with follow up performed within the Texas Children’s Hospital (TCH) healthcare system and compare this to current guidelines. Ultimately this work will help identify patient characteristics and recommend areas for reduced priority of intramuscular PCN usage in situations of drug shortage.

Result Tables

**Figure:**
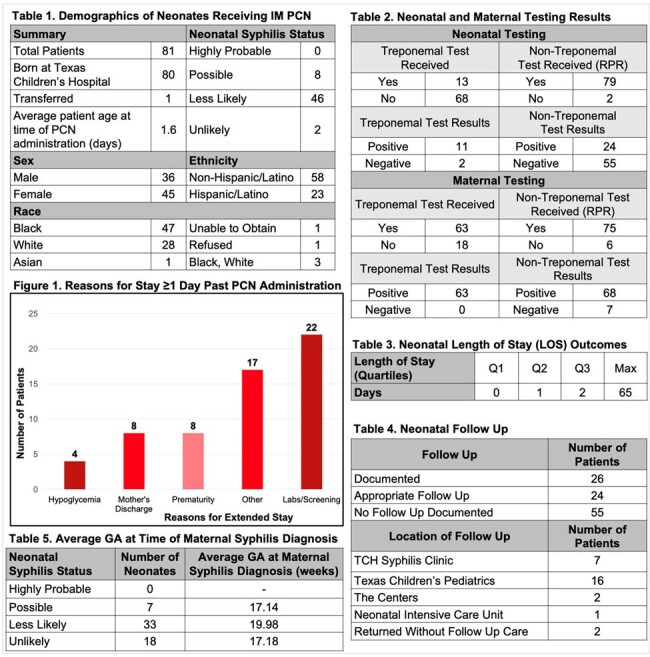

Demographic and Treatment Tables/Figures for infants who received treatment with IM benzathine Penicillin at birth.

**Methods:**

All infants who received a single dose of IM benzathine PCN between 08/2018 and 08/2023 were considered in this study. Demographic (race, ethnicity, sex, age), and treatment data (PCN administration and discharge dates, treponemal/nontreponemal test results for mother-infant dyad, location of follow up, and gestational age (GA) at time of maternal evaluation) were abstracted from the Epic® EHR.

**Results:**

Eighty-one infant received a single dose of IM benzathine PCN between 8/2018 and 8/2023. The average GA at maternal diagnosis was 18 weeks. Pending laboratory/screening results was the largest factor keeping neonates in the hospital 1 day or more past IM PCN administration. Premature infants were the largest population to remain hospitalized for greater than 10 days. Most neonates (71.6%) had a LOS greater than or equal to 1 day while 13 (16.0%) had a LOS of greater than or equal to 10 days. Twenty-six patients (32.1%) completed follow up within the TCH system with more than half being evaluated at TCH.

**Conclusion:**

Neonates with pending laboratory results, prematurity status, and mothers with extended stays should be further evaluated prior to IM PCN administration during times of shortage. Neonatal follow up screening could be improved with a dedicated syphilis clinic/screening program (such a clinic started at TCH in quarter 1 of 2022). Additional research is necessary to optimize inpatient/outpatient IM PCN use when treating congenital syphilis.

**Disclosures:**

**Ryan H. Rochat, MD, PhD, MS**, Merck Sharpe Dohme: Grant/Research Support

